# Radiographic and Clinical Results of Combined Bone and Soft-Tissue Tailored Surgeries for Hip Dislocation and Subluxation in Cerebral Palsy

**DOI:** 10.3390/children12010091

**Published:** 2025-01-15

**Authors:** Giulia Beltrame, Artemisia Panou, Andrea Peccati, Haridimos Tsibidakis, Francesco Pelillo, Nicola Marcello Portinaro

**Affiliations:** 1 Residency Program in Orthopaedic and Traumatology, University of Milan, 20122 Milan, Italy; 2 Orthopaedic Department, Penteli’s Children Hospital, 152 36 Penteli, Greece; 3 Department of Paediatric Surgery, Fondazione IRCCS Ca’ Granda Ospedale Maggiore Policlinico, 20122 Milan, Italy; 4 Second Orthopaedic Department, General Hospital G. Gennimatas, 115 27 Athens, Greece; 5 Orthopaedic Department, Policlinico San Matteo, 27100 Pavia, Italy

**Keywords:** cerebral palsy, hip dislocation, hip surgery

## Abstract

Background/Objectives: The aim of the study is to present middle-term results of tailored bone and soft-tissue surgeries in subluxated and dislocated hips in children affected by cerebral palsy. Methods: A total of 87 medical records belonging to 73 children affected by CP, treated with combined soft-tissue releases, VDO, and pelvic osteotomy, were reviewed retrospectively. Radiological measurements of AI, RI, and NSA were obtained before surgery, postoperatively, at 12 and 24 months after surgery. Results were assessed globally and by GMFCS, age, and Robin score. Results: Postoperative results are not statistically influenced by age and GMFCS levels at surgery. All three radiographic parameters showed persistent statistically significant improvement after surgery and at follow-up, respectively. Conclusions: Obtaining the best possible concentric reduction of the femoral head in the acetabulum, with simultaneous multilevel soft-tissue rebalancing, creates the best mechanical and biological environment to allow the reshaping of both articular surfaces, obtaining physiological internal joint pressure. The anatomical best congruency is protective from recurrence.

## 1. Introduction

Population-based studies have shown that patients affected by cerebral palsy (CP) have an increased risk of hip subluxation and dislocation [1], mainly due to spasticity-induced fixed muscle contractures and imbalance, responsible for acetabular and femoral bone deformities.

A direct correlation has been demonstrated between the occurrence of hip dislocation, measured as a Reimer migration percentage over 30% [2,3], and the patient’s level of mobility, according to the Gross Motor Function Classification System (GMFCS) [4]. Since hip subluxation precedes dislocation, its early clinical detection and monitoring are important. Radiological surveillance is recommended in all patients with CP, and it is mandatory if one and/or both hips present a reduced abduction and/or femoral shortening [5,6]. The cadence of the radiological surveillance should be tailored in accordance with the GMFCS level [7] as well as with the hip development [8], and a pelvis X-ray within 24 months of birth in severe bilateral cerebral palsy is strongly recommended [9].

The most important radiographic parameters are the Reimers Migration Index (RI), Acetabular Index (AI), and the proximal femoral Neck–Shaft Angle (NSA), all measured on standardized anterior-posterior pelvic radiographs.

The risk of hip displacement ranges from being very low at GMFCS level I up to 68–90% at GMFCS level V [10]. Treatment aims are vast as well; if the hip is subluxated, the aim is to prevent the dislocation; if the hip is dislocated, the aim might be to restore the correct anatomy and/or to remove pain. The treatment of choice depends on the patient’s age and the development of the hip [11]. It is noteworthy to mention that the persistence of valgus femur, anteversion, and acetabular dysplasia are well-known risk factors for hip instability, and they represent the target for early treatment [12,13]. The goal of surgical treatment is to create a stable and mobile joint to prevent osteoarticular degeneration and pain [11]. Soft-tissue surgery, as adductor and/or iliopsoas lengthening, can be performed when muscle–tendon contractures are present. When a severe subluxation is radiographically detectable, varus derotational osteotomy (VDO) is recommended to reduce soft-tissue tension surrounding the hip, improve the gluteus mechanical lever arm, and restore normal femoral anteversion [14]. Significant acetabular dysplasia, with RI > 50% and AI > 20°, requires pelvic osteotomy to ensure satisfactory hip coverage and stability. Several techniques have been described for the correction of acetabular dysplasia with good results over time. The aim of this study was to evaluate the outcomes of proximal femoral varus derotational osteotomy (VDRO) combined with pelvic osteotomy and selective soft-tissue releases for the treatment of subluxated and/or dislocated hips in children with cerebral palsy (CP). Radiographic outcomes were assessed at baseline, immediately postoperatively, and at 12 and 24 months follow-up. Additionally, the study aimed to examine the influence of patient age, functional level (per the GMFCS), and Robin’s score, which is a six-grade scale used to assess hip morphology, on these radiographic outcomes to determine the procedure’s effectiveness in achieving and maintaining hip stability [1].

## 2. Materials and Methods

Our study was conducted in accordance with the ethical standards of the Declaration of Helsinki (1964) and its subsequent amendments. Ethical approval was not required due to the retrospective nature of the study; however, the Hospital’s Institutional Review Board acknowledged the study. Written informed consent for the publication of patients’ clinical details was obtained from parents and caregivers, and a copy of the consent form is available for review by the Editor of this journal. This case series has been reported in compliance with the PROCESS Guideline.

We included patients aged 0–18 years diagnosed with CP, with GMFCS levels II–V and hips graded with Robin’s score I–V, who were treated between June 2007 and May 2019 with combined soft-tissue releases, VDO, and pelvic osteotomy (unilaterally or bilaterally) by the same highly specialized surgeons at the same hospital. 

The GMFCS level scale is a standardized tool used to classify a patient’s level of independence in mobility.

- Level I: Walks without limitations.
- Level II: Walks with limitations.
- Level III: Walks with a hand-held mobility device.
- Level IV: Limited self-mobility, often requiring powered mobility.
- Level V: Severe limitations in head and trunk control.

The Robin score is a six-grade scale used to assess hip morphology:

- Level I: Normal hip.
- Level II: Near-normal hip.
- Level III: Dysplastic hip.
- Level IV: Subluxated hip.
- Level V: Dislocated hip.
- Level VI: Hip requiring a salvage procedure.

Each included patient must provide at least 24 months of radiological follow-up. The Acetabular index (AI) was measured as the angle formed by Hilgenreiner’s line—a horizontal line passing through the triradiate cartilage—and the roof of the acetabulum. The Reimer’s Migration Index (RI) is the percentage of the femoral head located outside the acetabular bony margin. It was calculated as the ratio between the lateral uncovered portion of the femoral head and the total width of the femoral head. The inclusion of radiographic parameters for this study was based on key measurements essential for determining the appropriateness of the surgical interventions. Patients with a pre-operative AI of ≥20° and RI ≥ 50% were included. CT scans, including 3D reconstructions, are not routinely used for pre-operative planning in our practice, reserving it for cases where radiographs are insufficient to provide the necessary anatomical detail. Children with diagnoses other than CP, incomplete medical records, or those treated outside the study period or older than 18 years at the time of surgery were excluded. Exclusion radiographic parameters were pre-operative AI > 60°, as well as any patients showing radiographic evidence of advanced hip-joint degeneration or osteoarthritis. Many patients, particularly those with GMFCS IV and V, presented with pelvic obliquity, which was assessed preoperatively and considered during surgical planning, with pelvic osteotomies tailored to optimize acetabular coverage. While not systematically analyzed in this study, pelvic obliquity was an important factor in managing these complex cases.

The medical records of 87 hips belonging to 73 children were reviewed retrospectively.

Patients were classified according to their GMFCS level, while hips were classified according to the severity hip score described by Robin.

Femoral osteotomies were performed with a 90° blade steel plate. Periacetabular osteotomy, according to Pemberton, San Diego, or Dega, was performed depending on the acetabular defects using the bone graft obtained from the VDO wedge with no additional fixation. Simultaneous soft-tissue releases surrounding the hip, as adductor or psoas release, were performed to ease hip reduction. If the hip was not reduced after VDO and pelvic osteotomy, an opened reduction was considered to restore full joint congruency. Performing the open reduction after VDO and pelvic osteotomy allows us to accurately evaluate residual instability and address only what remains unresolved based on intra-operative fluoroscopy. In these cases, capsulorrhaphy is typically performed to tighten the joint capsule and enhance stability. Joint clearance, such as the removal of hypertrophic soft tissue or interposed fibrous tissue within the acetabulum, is carried out selectively when such obstructions are identified intraoperatively. After surgery, a knee–hip brace, fixed at 20° of flexion, was used for the following 6 weeks in all patients. Rehabilitation, including joint mobilization and soft-tissue stretching, started on the day after surgery, according to pain. Weight-bearing and/or standing were allowed at the first radiographic follow-up, 6 weeks after surgery.

**Figure 1:**
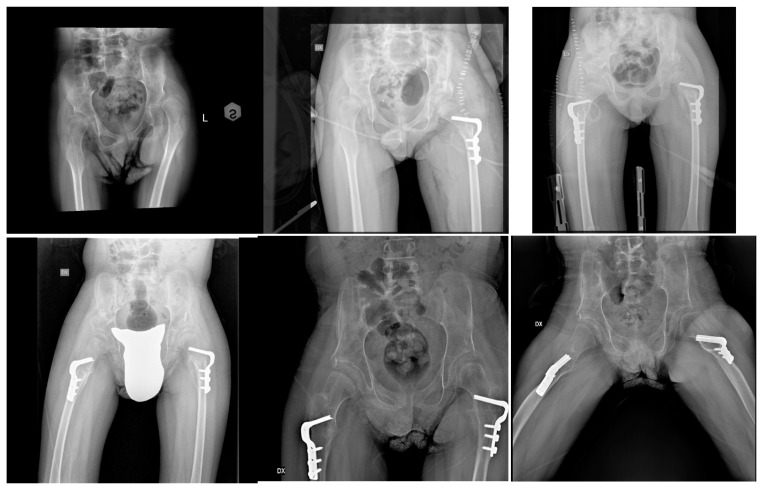
(**Top right**): bilateral femoral head and acetabular dysplasia in a 9-year-old boy GMFCS 4-pre-op; (**middle top**): immediate post-op of the left hip; (**top left**): immediate post-op of the right hip, three months apart from the left-sided surgery; (**bottom right**): follow-up at 12 months; (**middle bottom**) and (**left bottom**): post-op at 24 months. Note the good remodeling of the left femoral head.

Radiological measurements of AI, RI, and NSA were obtained before surgery, postoperatively, at 12 and 24 months after surgery; for each hip, the percentage variation between the 24-month follow-up and the pre-operative values of AI, RI, and NSA values were calculated (Figure 1). Complications of surgery during the whole follow-up period were recorded.

Data collection and analysis were performed using R/JAMOVI software (version 2.3.28). All measured and calculated values were tested for normality using the Shapiro–Wilk test and for homogeneity/homoscedasticity using Levene’s test, as these are fundamental assumptions for conducting ANOVA. When these assumptions were not met, the non-parametric Kruskal–Wallis test was used as an alternative to analyze the data. Mean values were calculated for all the patients and hips and then for patients and hips grouped by GMFCS level and by age (they were grouped in 0–4 y/o, 5–9 y/o, 10–14 y/o, or 15 y/o and older) and by Robin score, and means were compared using a paired Student’s *t*-test, ANOVA, or non-parametric Kruskal–Wallis test (*p* < 0.05 was considered to be statistically significant). Values were reported as mean ± SD or median/IQR.3.

## 3. Results

There were 50 males (68.5%) and 23 females (31.5%). According to GMFCS, 6 patients (8.2%) were classified as II, 14 (19.2%) as GMFCS III, 14 (19.2%) as GMFCS IV, and 25 (34.2%) as GMFCS V. For 14 patients (19.2%) GMFCS level was not available. According to Robin’s score, 2 hips (2.3%) were classified as grade I, 2 hips (2.3%) as grade II, 2 hips (2.3%) as grade III, 76 hips (87.4%) as grade IV, and 5 hips (5.7%) as grade V.

The mean age at surgery was 11.16 ± 3.09 years: one patient (1.4%) was aged 0–4, 29 patients (39.7%) were aged 5–9, 32 patients (43.8%) were aged 10–14 and 11 patients (15.1%) were aged 15 and above. A total of 65 patients (89.0%) were treated unilaterally, while 8 (11.0%) were treated bilaterally; 5 (62.50%) of these patients were treated with a single-stage surgery, and 3 (37.5%) of them with a two-stage surgery (mean of 176 ± 116 days between the first and the second procedure). For 72 hips (82.8%), combined VDO and pelvic osteotomy were performed at the same time, and for 29 hips (33.3%), soft-tissue procedures were associated, such as 19 adductor releases (73.08%), 4 capsular releases (15.38%), and 5 medial hamstrings releases (17.23). Psoas release was not needed in any case after the DVO. None of the patients had previously undergone hip surgery.

### 3.1. Acetabular Index (AI)

**Figure 2:**
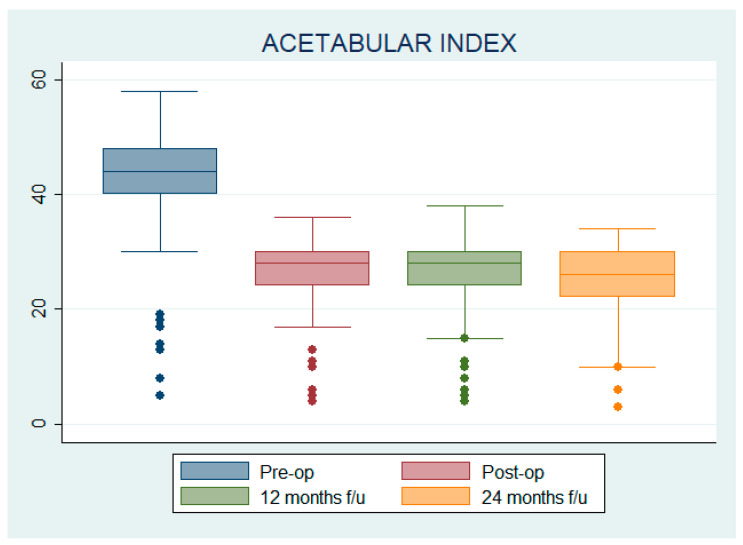
Acetabular index pre-operative, postoperative, at 12 months follow-up, and at 24 months follow-up.

The mean pre-operative AI was 41.91 ± 10.70°, while the mean postoperative AI was 25.77 ± 7.50°; at 12 and 24 months after surgery, AI was 25.66 ± 7.61° and 24.74 ± 7.47°, respectively. No statistically significant differences in AI at any time were found when patients and hips were grouped by age, GMFCS level, or Robin’s score. All the variations in AI were statistically significant compared to pre-op; the mean difference with respect to pre-op was 16.14 ± 0.73° (IC 14.69–17.59) for post-op, 16.25 ± 0.78° (IC 14.70–17.81) for 12-months follow-up and 17.17 ± 0.76° (IC 15.66–18.68) for 24-months follow-up (see Figure 2).

The mean AI percentage variation between the 24-month follow-up and the pre-operative AI was −0.54 ± 0.15 for GMFCS II patients, −0.39 ± 0.19 for GMFCS III patients, −0.39 ± 0.12 for GMFCS IV patients, −0.41 ± 0.13 for GMFCS V patients and −0.38 ± 0.07 for those patients whose GMFCS level was not available.

The mean AI percentage variation between the 24-month follow-up and the pre-operative AI was −0.46 ± 0.11 for Robin I hips, −0.39 ± 0.10 for Robin 2 hips, −0.41 ± 0.04 for Robin 3 hips, −0.41 ± 0.14 for Robin 4 hips, and −0.36 ± 0.14 for Robin 5 hips.

The mean AI percentage variation between the 24-month follow-up and the pre-operative AI was −0.40 ± 0.13 for patients aged under 10, −0.42 ± 0.15 for patients aged 11–15, −0.36 ± 0.09 for patients aged over 15.

Since AI percentage variations were not normally distributed (W = 0.903; *p* < 0.001), Kruskal–Wallis test was used, showing no statistically significant differences in the mean percentage variation of AI at the 24-month follow-up compared to pre-operative AI among GMFCS levels (Chi^2^ = 7.08, *p* = 0.13, df = 4), Robin’s score (Chi^2^ = 1.55; *p* = 0.82; df = 4), or patients’ age (Chi^2^ = 1.35, *p* = 0.51, df = 2).

### 3.2. Reimer’s Index (RI)

The mean pre-operative RI was 62.99 ± 23.90%, while the mean postoperative RI was 3.40 ± 3.97%; at 12 and 24 months after surgery, RI was 4.11 ± 4.96% and 4.09 ± 4.91%, respectively. Statistically significant differences in RI were found according to GMFCS level only before surgery (*p* < 0.01) and according to age only before surgery (*p* = 0.03) and at 12-month follow-up (*p* = 0.03).

**Figure 3:**
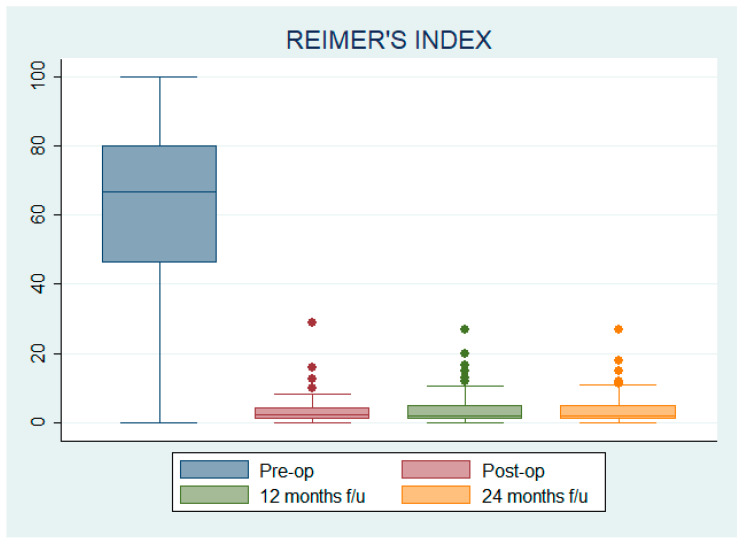
Reimer index pre-operative, postoperative, at 12 months follow-up, and at 24 months follow-up.

All the variations in RI were statistically significant compared to pre-op; the mean difference with respect to pre-op was 59.59 ± 2.59% (IC 54.45–64.73) for post-op, 58.88 ± 2.64% (IC 53.64–64.12) for 12-month follow-up, and 58.90 ± 2.62% (IC 53.70–64.10) for 24-month follow-up (see Figure 3).

The RI mean percentage variation between the 24-month follow-up and the pre-operative was −0.98 ± 0.02 for GMFCS II patients, −0.88 ± 0.20 for GMFCS III patients, −0.91 ± 0.10 for GMFCS IV patients, −0.95 ± 0.05 for GMFCS V patients, and −0.93 ± 0.08 for those patients whose GMFCS level was not available.

The mean RI percentage variation between the 24-month follow-up and the pre-operative AI was −0.92 ± 0.10 for Robin I hips, −0.97 ± 0.01 for Robin 2 hips, −0.91 ± 0.11 for Robin 3 hips, −0.93 ± 0.11 for Robin 4 hips, and −0.92 ± 0.07 for Robin 5 hips.

The mean RI percentage variation between the 24-month follow-up and the pre-operative RI was −0.90 ± 0.14 for patients aged under 10, −0.95 ± 0.05 for patients aged 11–15, and −0.94 ± 0.07 for patients aged over 15.

Since RI percentage variations were not normally distributed (W = 0.605; *p* < 0.001), the Kruskal–Wallis test was used, showing no statistically significant differences in the mean percentage variation of AI at the 24-month follow-up compared to pre-operative AI among GMFCS levels (Chi^2^ = 4.96, *p* = 0.29, df = 4), Robin score (Chi^2^ = 1.68; *p* = 0.79; df = 4), or patients’ age (Chi^2^ = 4.19; *p* = 0.12; df = 2).

### 3.3. Neck–Shaft Angle (NSA)

The mean pre-operative NSA was 161.81 ± 7.84°, while the mean postoperative NSA was 121.52 ± 10.81°; at 12 and 24 months after surgery, NSA was 121.61 ± 10.24° and 122.20 ± 8.74°, respectively. Statistically significant differences in NSA were found according to patients’ age GMFCS level only before surgery (*p* = 0.04).

**Figure 4:**
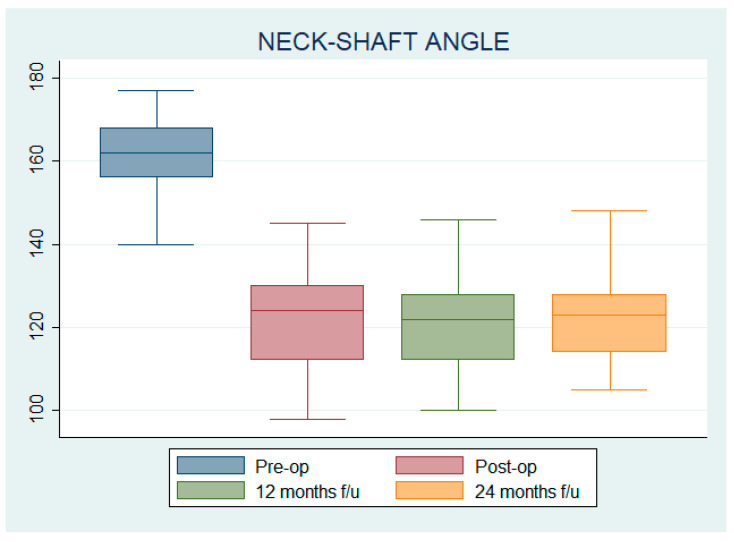
Neck–shaft angle pre-operative, postoperative, at 12 months follow-up, and at 24 months follow-up.

All the variations in NSA were statistically significant compared to pre-op; the mean difference with respect to pre-op was 40.29 ± 1.32° (IC 37.66–42.92) for post-op, 40.20 ± 1.17° (IC37.86–42.53) for 12-month follow-up, and 39.61 ± 1.11° (IC 37.41–41.81) for 24-month follow-up (see Figure 4).

The NSA mean percentage variation between the 24-month follow-up and the pre-operative NSA was −0.25 ± 0.05 for GMFCS II patients, −0.23 ± 0.07 for GMFCS III patients, −0.25 ± 0.06 for GMFCS IVand V patients, and −0.24 ± 0.06 for those patients whose GMFCS level was not available.

The mean NSA percentage variation between the 24-month follow-up and the pre-operative NSA was −0.19 ± 0.10 for Robin I hips, −0.24 ± 0.00 for Robin 2 hips, −0.25 ± 0.05 for Robin 3 hips, −0.25 ± 0.06 for Robin 4 hips, and −0.22 ± 0.04 for Robin 5 hips.

The mean NSA percentage variation between the 24-month follow-up and the pre-operative RI was −0.24 ± 0.05 for patients aged under 10, −0.25 ± 0.06 for patients aged 11–15, and −0.24 ± 0.05 for patients aged over 15.

Since NSA percentage variations were normally distributed (W = 0.984; *p* = 0.362) and homogeneity was not violated (Levene’s test, F = 0.344, *p* = 0.847), one-way ANOVA was used to show no statistically significant differences in the mean percentage variation of NSA at the 24-month follow-up compared to pre-operative AI among GMFCS levels (F = 0.571, *p* = 0.69, df = 4), Robin score (F = 0.668, *p* = 0.62; df = 4) or patient age (F = 0.173, *p* = 0.84, df = 84).

### 3.4. Complications

Early complications (<30 days after surgery) were recorded in 2 hips (2.3%):

- blood transfusion in 1 case (1.1%);
- wound dehiscence in 1 case (1.1%).

Late complications (>30 days after surgery) were recorded in 12 hips (13.8%):

- hip dislocation in 6 cases (6.9%), requiring new surgery (in 84 days);
- graft resorption in 3 cases (3.4%), requiring new surgery (in 6 days) only in one case;
- infection in 1 case (1.1%), requiring removal of metal;
- hardware failure required re-surgery (in 2 days) in one case (1.1%);
- uncontrolled pain for more than 6 months from surgery in one case (1.1%), with complete resolution at the latest follow-up.

No avascular necrosis of the femoral head, iatrogenic fracture of the femur, chondrolysis, osteolysis, premature closure of triradiate cartilage, or hardware loosening was recorded.

## 4. Discussion

Children suffering from CP present an increased risk of hip subluxation and/or dislocation, with a relative risk ranging from 1 to 75%, in accordance with the severity of motor impairment and the level of mobility, as classified by the GMFCS level. In severe cases (GMFCS III, IV, and V), soft-tissue releases and surgical bony procedures are required [15]. Among surgeries, minimally invasive procedures such as temporary proximal femoral epiphysiodesis have been shown to prevent further femoral head subluxation [13]; femoral and/or acetabular osteotomy otherwise aims at restoring the normal anatomy biomechanics of the hip joint [16]. Multilevel soft-tissue lengthening reduces muscle–tendon contractures responsible for spasticity-induced acetabular and femoral deformity [15]. In our unit, VDO was indicated in ambulatory and non-ambulatory patients, presenting with femoral anteversion associated with coxa valga and acetabular mild dysplasia, aiming at correcting the Lever Arm Dysfunction (LAD) [17]. Routinely pre-operative AP pelvic X-rays were taken to assess RI, AI, and NSA. AI has been identified in the literature as the most powerful single radiographic predictor for hip displacement, but it is not a prognostic indicator below the age of 5 years and depends on pelvic rotation, flexion/extension, and lateral tilt [18,19]. On the other hand, migration percentage is of prognostic value for the development of hip displacement, as it is not influenced by the rotational malposition of the femur [20]. The Neck–Shaft Angle (NSA) depends as well on femoral rotational profiles with greater intra- and inter-observer errors. The patient-specific surgical plan was also refined at the time of surgery with a fluoroscopic evaluation of the femoral head and acetabular best position for congruency. More than 50% of patients were GMFCS level IV or V, and the majority experienced deterioration in the morphology of both hips using the classification described by Robin et al. [21] with progression to subluxation and frank dislocation. The study aimed to assess whether patient age and functional level (as categorized by GMFCS) at the time of surgery impacted radiographic outcomes. Differently from the literature, the results showed that age and GMFCS levels had no statistically significant effect on postoperative improvements or the maintenance of stability over time [16]. This finding suggests that the surgical approach is broadly effective for pediatric patients with CP, regardless of age or functional level. The stability achieved through selective soft-tissue release and VDO combined with pelvic osteotomy appears consistent, and the outcomes seem primarily driven by the surgical technique itself. If the AI was less than 25°, soft-tissue release and VDO were initially performed; in the case of persistent instability and/or inadequate femoral head coverage, pelvic osteotomy and open reduction were performed to achieve maximum congruency of the joint [8,14]. In borderline cases, previous authors reported that combined femoral and pelvic osteotomies improve femoral head coverage, reducing pain and increasing ROM [14,22,23,24]. It should be mentioned that these surgical procedures were well tolerated by most of the patients, as demonstrated in the literature [25]. The rate of early complications in our study was 2.3%, while late complications were recorded in 12 hips (13.8%). The cases of hip dislocation had a pre-operative RI over 50% (one of them of 100%), identified as a risk factor for a high failure rate, including re-dislocation of the operated hip or displacement of the contralateral hip at skeletal maturity [21]. Good remodeling of the femoral head, even in older patients, was detected with no avascular necrosis of the femoral head even with head deformity Graham type 5b, in accordance with Min et al. [26]. Our findings suggest that the positive outcomes observed are likely due to our meticulous approach to achieving an optimal concentric reduction of the femoral head within the acetabulum, along with precise anatomical alignment to encourage reshaping of both articular surfaces. When needed, femoral shortening helps restore the natural 3-mm femoral head tilt within the acetabular socket, as verified by fluoroscopy. Combined with thorough multilevel soft-tissue balancing, this approach creates an ideal mechanical and biological setting that promotes acetabular convexity and femoral head sphericity while reducing joint pressure. Nonetheless, it is important to notice that postoperative success also depends on appropriate physical therapy, maintaining an upright position, and avoiding scissoring of the legs, requiring a multidisciplinary approach to optimize outcomes and sustain the benefits of surgical intervention.

Despite the lack of quality-of-life measures or patient-reported outcomes in this study, we believe that the low rate of complications, combined with the observed radiographic improvements, strongly suggests that these surgical interventions positively impact patients’ daily lives. By achieving stable joint alignment and restoring hip congruency, these procedures help reduce pain, improve range of motion, and establish a structural foundation essential for functional improvements.

Limitations of this study include its short-term follow-up and the absence of quality-of-life measures, which were outside our study’s primary focus on radiographic outcomes. Despite this limitation, we believe the surgical interventions studied are highly beneficial for patients with CP. A prospective, multicenter study with longer follow-up and patient-reported outcomes would provide more comprehensive insight.

## 5. Conclusions

In conclusion, we recommend performing early VDRO besides the level of disability and head deformity associated with acetabuloplasty when AI is more than 30°, or the femoral head is dislocated and deformed at any age of the patients. Simultaneous soft-tissue release is indicated when tension is detected after bony procedures to prevent extra pressure on the hip joint and decrease the risk of recurrence. Pre-operative MP and AI can assist with surgical planning, but an approach determined by the intra-operative congruency of the joint is necessary to determine the optimum surgical treatment. A longer follow-up assessment would help to evaluate whether these positive outcomes are sustained over time, further solidifying the durability of our approach.

## Data Availability

The data presented in this study are available on request from the corresponding author. The data are not publicly available due to privacy reasons.
